# Resting heart rate and incident atrial fibrillation: A stratified Mendelian randomization in the AFGen consortium

**DOI:** 10.1371/journal.pone.0268768

**Published:** 2022-05-20

**Authors:** J. E. Siland, B. Geelhoed, C. Roselli, B. Wang, H. J. Lin, S. Weiss, S. Trompet, M. E. van den Berg, E. Z. Soliman, L. Y. Chen, I. Ford, J. W. Jukema, P. W. Macfarlane, J. Kornej, H. Lin, K. L. Lunetta, M. Kavousi, J. A. Kors, M. A. Ikram, X. Guo, J. Yao, M. Dörr, S. B. Felix, U. Völker, N. Sotoodehnia, D. E. Arking, B. H. Stricker, S. R. Heckbert, S. A. Lubitz, E. J. Benjamin, A. Alonso, P. T. Ellinor, P. van der Harst, M. Rienstra

**Affiliations:** 1 Department of Cardiology, University of Groningen, University Medical Center Groningen, Groningen, The Netherlands; 2 Cardiovascular Disease Initiative, The Broad Institute of MIT and Harvard, Cambridge, MA, United States of America; 3 Department of Biostatistics, Boston University School of Public Health, Boston, MA, United States of America; 4 Institute for Translational Genomics and Population Sciences, The Lundquist Institute at Harbor-UCLA Medical Center, Torrance, CA, United States of America; 5 Department of Pediatrics, David Geffen School of Medicine at UCLA, Los Angeles, CA, United States of America; 6 Interfaculty Institute for Genetics and Functional Genomics; Department of Functional Genomics; University Medicine Greifswald, Greifswald, Germany; 7 DZHK (German Centre for Cardiovascular Research); partner site Greifswald, Greifswald, Germany; 8 Department of Cardiology, Leiden University Medical Center, Leiden, The Netherlands; 9 Department of Internal Medicine, Section of Gerontology and Geriatrics, Leiden University Medical Center, Leiden, The Netherlands; 10 Department of Epidemiology, Erasmus University Medical Center Rotterdam, Rotterdam, The Netherlands; 11 Division of Public Health Sciences and Department of Medicine, Epidemiological Cardiology Research Center, Department of Epidemiology and Prevention, Section on Cardiology, Wake Forest School of Medicine, Winston-Salem, NC, United States of America; 12 Cardiovascular Division, Department of Medicine, University of Minnesota Medical School, Minneapolis, MN, United States of America; 13 Robertson Centre for Biostatistics, University of Glasgow, Glasgow, United Kingdom; 14 Einthoven Laboratory for Experimental Vascular Medicine, LUMC, Leiden, The Netherlands; 15 Netherlands Heart Institute, Utrecht, The Netherlands; 16 Institute of Health and Wellbeing, College of Medical, Veterinary and Life Sciences, University of Glasgow, Glasgow, United Kingdom; 17 National Heart, Lung, and Blood Institute’s and Boston University’s Framingham Heart Study, Framingham, MA, United States of America; 18 National Heart Lung and Blood Institute’s and Boston University’s Framingham Heart Study, Framingham, MA, United States of America; 19 Section of Computational Biomedicine, Department of Medicine, Boston University School of Medicine, Boston, MA, Unites States of America; 20 Department of Medical Informatics, Erasmus University Medical Center Rotterdam, Rotterdam, The Netherlands; 21 Institute for Translational Genomics and Population Sciences, Department of Pediatrics, The Lundquist Institute at Harbor-UCLA Medical Center, Torrance, CA, United States of America; 22 Department of Internal Medicine B-Cardiology, Pneumology, Infectious Diseases, Intensive Care Medicine, University Medicine Greifswald, Greifswald, Germany; 23 Cardiovascular Health Research Unit, Division of Cardiology, Departments of Medicine and Epidemiology, University of Washington, Seattle, WA, Unites States of America; 24 McKusick-Nathans Institute, Department of Genetic Medicine, Johns Hopkins University SOM, Baltimore, MD, Unites States of America; 25 Cardiovascular Health Research Unit and the Department of Epidemiology, University of Washington, Seattle, WA, Unites States of America; 26 Cardiovascular Research Center, Massachusetts General Hospital, Boston, MA, Unites States of America; 27 Cardiac Arrhythmia Service, Massachusetts General Hospital, Boston, MA, Unites States of America; 28 Department of Medicine, Boston University School of Medicine, Boston, MA, Unites States of America; 29 Department of Epidemiology, Boston University School of Public Health, Boston, MA, Unites States of America; 30 Department of Epidemiology, Rollins School of Public Health, Emory University, Atlanta, GA, Unites States of America; 31 University of Groningen, University Medical Center Groningen, Department of Genetics, Groningen, The Netherlands; 32 University Medical Center Utrecht, Department of Heart and Lungs, University of Utrecht, Utrecht, The Netherlands; University of Minnesota, UNITED STATES

## Abstract

**Background:**

Both elevated and low resting heart rates are associated with atrial fibrillation (AF), suggesting a U-shaped relationship. However, evidence for a U-shaped causal association between genetically-determined resting heart rate and incident AF is limited. We investigated potential directional changes of the causal association between genetically-determined resting heart rate and incident AF.

**Method and results:**

Seven cohorts of the AFGen consortium contributed data to this meta-analysis. All participants were of European ancestry with known AF status, genotype information, and a heart rate measurement from a baseline electrocardiogram (ECG). Three strata of instrumental variable-free resting heart rate were used to assess possible non-linear associations between genetically-determined resting heart rate and the logarithm of the incident AF hazard rate: <65; 65–75; and >75 beats per minute (bpm). Mendelian randomization analyses using a weighted resting heart rate polygenic risk score were performed for each stratum.

We studied 38,981 individuals (mean age 59±10 years, 54% women) with a mean resting heart rate of 67±11 bpm. During a mean follow-up of 13±5 years, 4,779 (12%) individuals developed AF. A U-shaped association between the resting heart rate and the incident AF-hazard ratio was observed. Genetically-determined resting heart rate was inversely associated with incident AF for instrumental variable-free resting heart rates below 65 bpm (hazard ratio for genetically-determined resting heart rate, 0.96; 95% confidence interval, 0.94–0.99; p = 0.01). Genetically-determined resting heart rate was not associated with incident AF in the other two strata.

**Conclusions:**

For resting heart rates below 65 bpm, our results support an inverse causal association between genetically-determined resting heart rate and incident AF.

## Introduction

Resting heart rate is a known predictor of several cardiovascular conditions, such as myocardial infarction, heart failure, and stroke [1–4]. For some conditions, including heart failure, lower heart rates can be associated with event reduction, providing evidence that heart rate may be a modifiable, causal risk factor—and not just a risk marker or a reflection of comorbidities [5–7]. Heart rate control is also an effective strategy for improving symptoms and reducing the risk of adverse cardiovascular events related to atrial fibrillation (AF) [8]. Several epidemiological studies have suggested that resting heart rate is a risk factor for development of AF. However, the shape of the association is unclear. While only lower heart rates were associated with an increased risk of incident AF in some studies [9–11], other studies found that an elevated resting heart rate were also associated [12]. A U-shaped association has been reported [13,14].

The pathways underlying the association between resting heart rate and incident AF are not fully understood. Resting heart rate is regulated by complex interactions of biological systems, including the autonomic nervous system. Some of the genetic loci associated with resting heart rate are related to arrhythmia susceptibility [1,15]. Recently, a Mendelian randomization analysis was performed in an attempt to determine if genetic loci that affect resting heart rate are causal to AF. The study inferred an inverse causal linear association between resting heart rate and incident AF in 367,703 individuals (including 13,538 AF cases) in the UK Biobank [16]. However, U-shaped associations were not investigated, because Mendelian randomization analyses allow testing of only linear associations.

Mendelian randomization can be viewed as randomized controlled trials, with genotypes randomly assigned at birth. Therefore, an association between genetic variants that determine resting heart rate and incident AF implies causality, assuming that genetically-determined resting heart rate is not directly associated with possible confounding factors.

Evidence for a U-shaped causal association between genetically-determined resting heart rate and incident AF may provide insight into how AF is initiated, and may lead to improved AF risk assessment. We divided resting heart rate into three strata to observe potential directional changes of the linear associations involved in Mendelian randomization analyses. The approach allowed us to investigate evidence for a U-shaped causal association.

## Methods

### Study population

**Fig 1:**
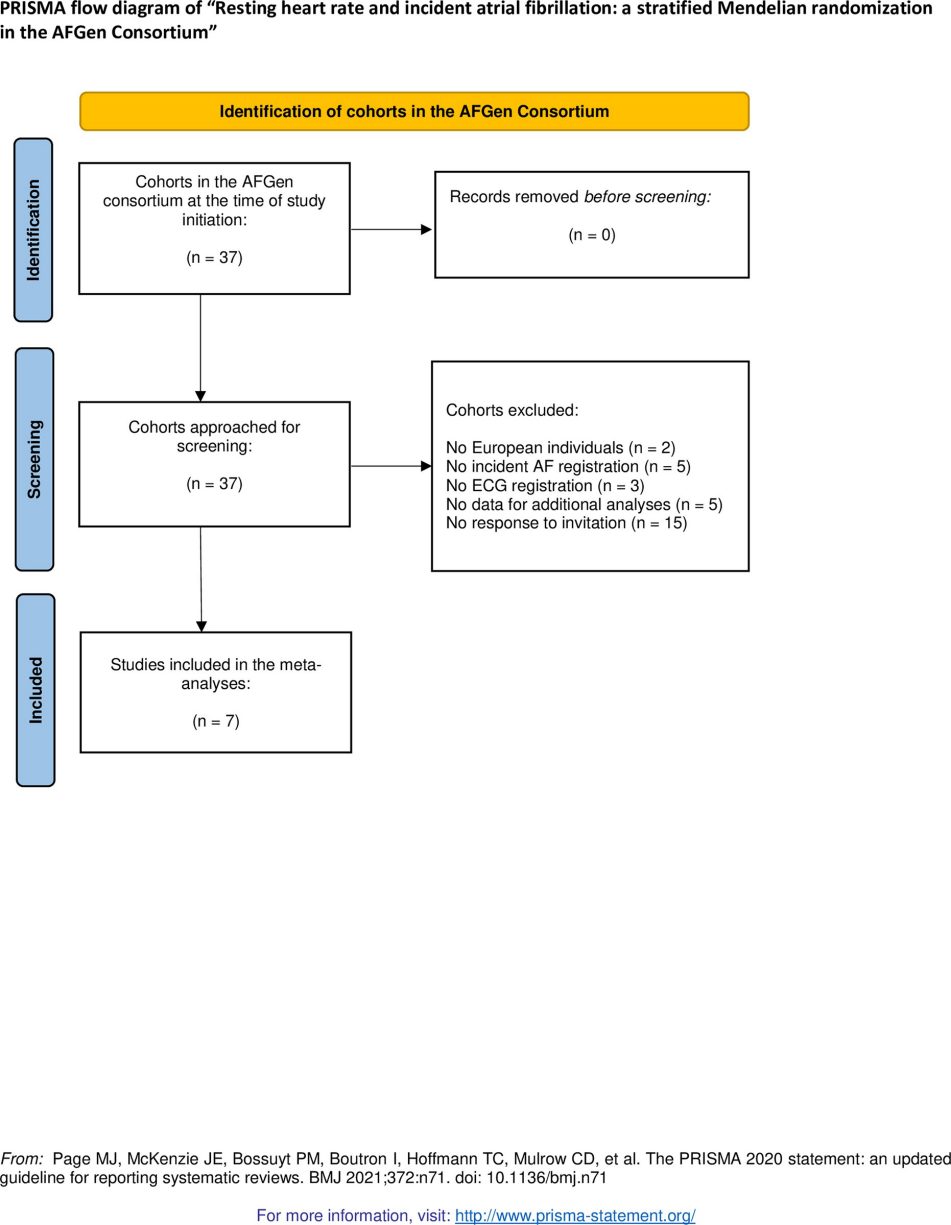
PRISMA flow diagram.

The meta-analysis was performed in seven cohorts of the AFGen consortium: the Atherosclerosis Risk in Communities (ARIC) study; the Framingham Heart Study (FHS); the Multi-Ethnic Study of Atherosclerosis (MESA); the Prevention of Renal and Vascular End-stage Disease (PREVEND) study; the PROspective Study of Pravastatin in the Elderly at Risk (PROSPER) study; the Rotterdam Study (RS-I and RS-II); and the Study of Health in Pomerania (SHIP). The selection of the cohorts is visualized in **Fig 1**. Detailed cohort descriptions are referenced in the **Supplementary Notes in** **S1 File**.

Participants were of European ancestry, with known AF status and a resting heart rate measured at baseline with a 10-second 12-lead electrocardiogram (ECG). Individuals with prevalent AF at baseline were excluded. We did not exclude individuals with negative dromotropic medication or pacemakers. Additionally, genome-wide genotyping was performed for the common genetic variants used in the resting heart rate polygenic risk score (PRS) (**S1 Table** in **S1 File**). Written informed consent by all participants and approval of the ethical review boards of all participating cohorts were provided and specifications can be found in the referenced cohort descriptions in the supplementary data.

### Ascertainment of atrial fibrillation

AF was assessed by each cohort, as referenced in the **supplementary notes in S1 File**. The AF status of participants was established by evidence on follow-up ECG recordings and/or by diagnostic codes for AF [International Statistical Classification of Diseases and Related Health Problems (ICD)].

### Assessment of resting heart rate and comorbidities

Resting heart rate was measured as a continuous covariate by a resting ECG recorded during baseline evaluations. Information was collected on recognized AF risk factors, such as hypertension, diabetes, heart failure, myocardial infarction and body mass index (BMI). Age was set as age at the time of the baseline ECG. Follow-up duration was defined as the time between the baseline ECG and diagnosis of incident AF or censoring. Detailed information is described for each cohort in the **Supplementary Notes in S1 File**.

### Genotyping and genetic instrument

Genotyping methodologies within the AFGen consortium were described previously [17]. A weighted PRS was calculated for each participant, incorporating genetic variants previously associated with resting heart rate [1]. Specifically, the PRS was the sum of the dose of each effect allele multiplied by the allele effect size (beta coefficient) (listed in **S1 Table** in **S1 File**). The heart rate PRS was used as the genetic instrument in the Mendelian randomization. Pleiotropy was assessed by adjusting for heart rate in the regression analyses of resting heart rate PRS and incident AF.

### Statistical analysis

Mendelian randomization analyses were stratified for instrumental variable-free resting heart rate. The instrumental variable-free heart rate is the heart rate without the effect of the heart rate PRS [18]. First, multivariable-adjusted Cox proportional hazard regressions of resting heart rate as a risk factor for incident AF were performed in each cohort. However, no time to event data was available for one of the cohorts (SHIP), for which logistic regression was performed. The odds ratio from SHIP was used as an approximation of the hazard ratio and meta-analyzed with the other hazard ratios.

Heart rate was included in the models of the regressions using linear and quadratic terms together with age, sex, eigen vector, and center if appropriate. From the beta-coefficients of the linear and quadratic terms, the hazard ratio (HR) and resting heart rate were derived for each cohort. Meta-analysis of the beta-coefficients of the linear and quadratic terms were used to derive a meta-analyzed value of the HR and resting heart rate.

**Fig 2:**
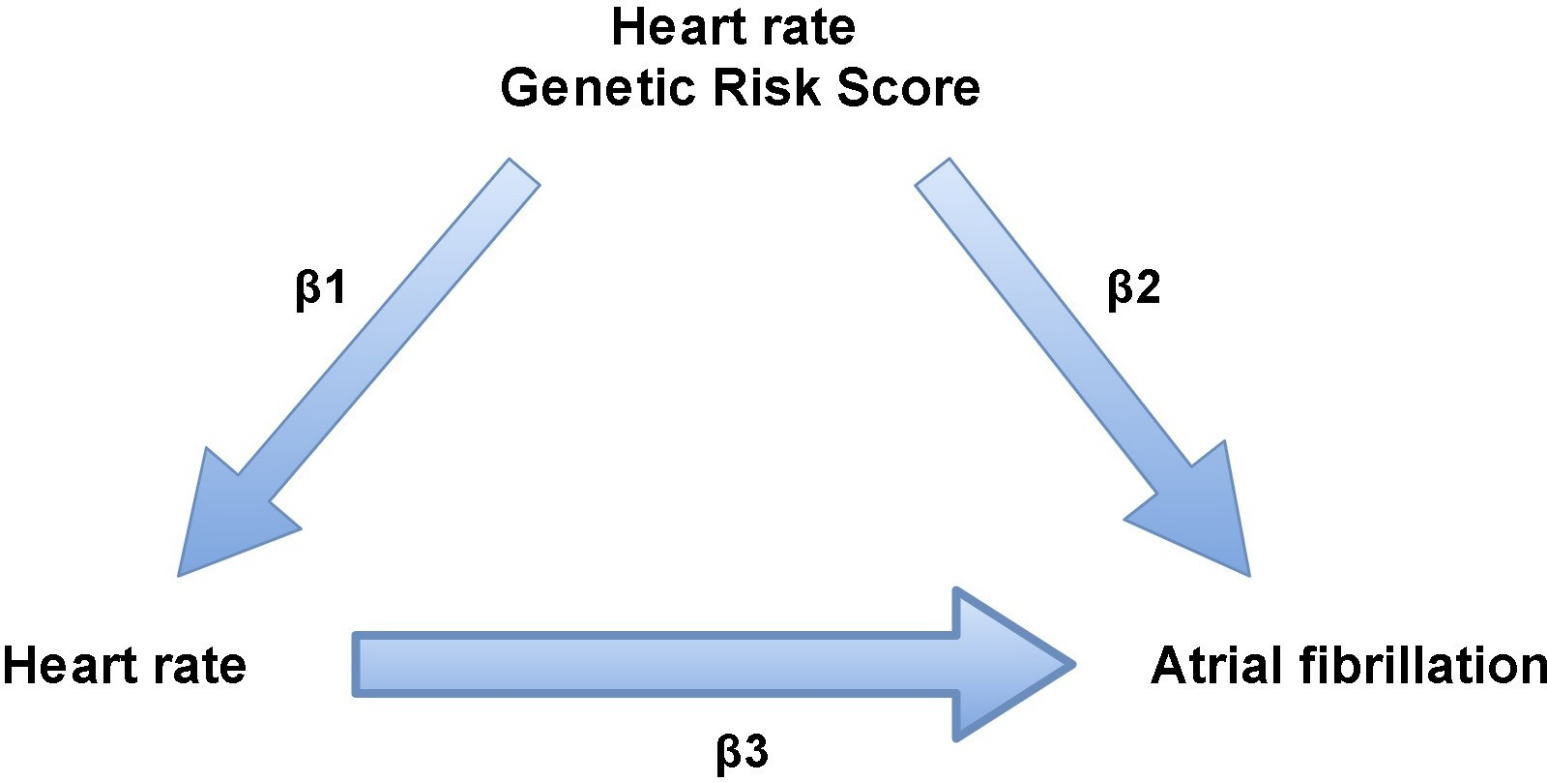
Visualization of the ratio estimator.

The association between genetically-determined resting heart rate and incident AF was investigated by performing Mendelian randomization studies in three strata, with approximately equal numbers of individuals in the three groups of heart rate. The instrumental variable-free heart rate distribution is used to stratify heart rate and avoid an association between heart rate PRS and incident AF based on the association between the heart rate PRS and heart rate [18]. The genetic variants were tested for pleiotropic effects by performing a regression between resting heart rate PRS and incident AF, adjusted for heart rate. A significant result suggests pleiotropy. We used the ratio method of Mendelian randomization. Specifically, the causal beta is estimated as the ratio of (1) a beta-coefficient representing the association between resting heart rate PRS and incident AF to (2) a beta coefficient representing the association between the heart rate PRS and resting heart rate. The resulting beta coefficients, when exponentiated, express the causal hazard ratio of AF per bpm increase in resting heart rate (**Fig 2**). The standard error of the three causal beta coefficients was calculated by use of Taylor series expansion [19]. Bonferroni corrected statistical significance was defined as p < 0.017 based on testing three strata. All analyses were performed using the R package (version 3.1.6; R Foundation for Statistical Computing, Vienna, Austria). Meta-analyses were performed with the function metagen () of the meta R package. Forest plots were created with the forest () function of the metaphor R package. Cox regressions were performed with the coxph function () of the survival R package.

First, regression analyses of resting heart rate PRS and instrumental variable-free resting heart rate (β_1_) and regression analyses of resting heart rate PRS and incident AF (β_2_) are calculated using the following formula’s; $\beta_{1}=\frac{\Delta Resting\;heart\;rate}{\Delta Resting\;heart\;rate\;PRS}$ and $\beta_{2}=\frac{\Delta \log\;hazard\;rate\;of\;Incident\;AF}{\Delta Resting\;heart\;rate\;PRS}$. Beta coefficients of three resting heart rate strata were calculated separately. Subsequently, $\beta_{3}=\frac{\beta 2}{\beta 1}$ was calculated for each stratum. The hazard ratio (ℯ ^β3^) of each stratum indicates the causal association between resting heart rate and incident AF. Abbreviations: PRS = Polygenic risk score, HR = Hazard ratio.

## Results

### Population

A total of 38,981 individuals were studied (mean age 59±10 years, 54% women). The mean resting heart rate was 67±11 bpm on baseline ECGs. A total of 4,779 (12%) individuals developed AF during a mean follow-up period of 13±5 years. **Table 1** shows baseline characteristics of participating cohorts.

**Table 1:** Characteristics of individuals of European ancestry included in the participating cohorts of AFGen.

Characteristics	ARIC (n = 8994)	FHS (n = 7829)	MESA (n = 2489)	PREVEND (n = 3501)	PROSPER (n = 5244)	RS I (n = 5043)	RS II (n = 1987)	SHIP (n = 3894)
**Age in years**	54 ± 6	53 ± 16	63 ± 10	49 ± 12	75 ± 3	68 ± 9	65 ± 8	49 ± 16
**Male**	4214 (47)	3553 (45)	1183 (48)	1804 (52)	2524 (48)	2021 (40)	899 (45)	1908 (49)
**Resting heart rate in bpm**	67 ± 10	64 ± 11	63 ± 10	69 ± 10	66 ± 12	71 ± 12	69 ± 11	73 ± 12
**Hypertension**	2393 (27)	2567 (33)	957 (38)	972 (28)	3257 (62)	1802 (36)	794 (40)	2017 (52)
**Diabetes**	763 (9)	489 (7)	146 (9)	133 (4)	544 (10)	517 (10)	213 (11)	413 (11)
**BMI in kg/m^2^**	27.0 ± 4.9	27.3 ± 5.4	27.7 ± 5.1	26.2 ± 4.3	26.8 ± 4.2	26.4 ± 3.9	27.3 ± 4.1	27.3 ± 4.8
**Heart failure**	309 (3)	85 (1)	0 (0)	6 (0.2)	0 (0)	126 (3)	22 (1)	305 (8)
**Myocardial infarction**	413 (5)	186 (2)	0 (0)	86 (3)	708 (14)	255 (5)	73 (4)	102 (3)
**Incident AF**	1817 (20)	757 (10)	448 (7)	169 (5)	505 (10)	818 (16)	171 (9)	94 (2)
**Follow-up duration in years**	22 ± 7	11 ± 4	12 ± 4	11 ± 3	3 ± 1	15 ± 8	12 ± 4	NA*

Values are mean ± standard deviation or N (%). Abbreviations: AF = atrial fibrillation, ARIC = Atherosclerosis Risk in Communities study, BMI = body mass index, bpm = beats per minute, ECG = electrocardiogram, FHS = Framingham Heart Study, MESA = Multi-Ethnic Study of Atherosclerosis, PREVEND = Prevention of Renal and Vascular End-stage Disease study, PROSPER = PROspective Study of Pravastatin in the Elderly at Risk study, RS = Rotterdam Study, SHIP = Study of Health in Pomerania. * Since no time to event (event = incident AF) was available, linear regressions analyses were performed for the SHIP cohort.

### Resting heart rate and atrial fibrillation

**Fig 3:**
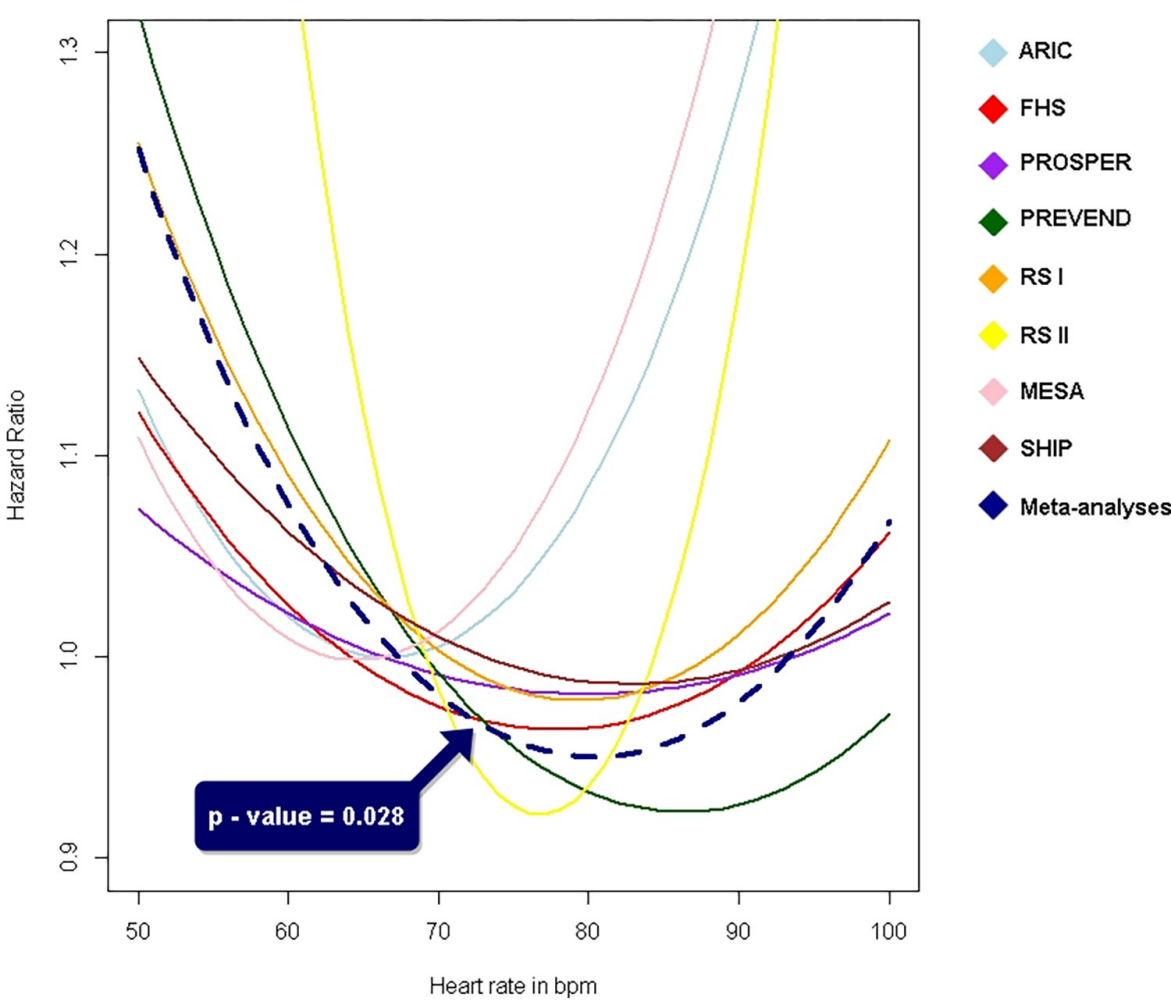
U-shaped association between resting heart rate and incident AF in seven cohorts of the AFGen consortium. A regression analysis using a quadratic term was performed per cohort to explore a non-linear association. The meta-analysed quadratic term of the association between resting heart rate and incident AF is significant (p-value = 0.028).

Observed associations between resting heart rate and incident AF are plotted in **Fig 3**. The meta-analyzed association between resting heart rate and incident AF showed a U-shaped curve (p = 0.028). Thus, both lower and higher resting heart rates were positively associated with incident AF.

### Resting heart rate and atrial fibrillation: Causal inference using the ratio estimator

The heart rate PRS was associated with resting heart rate in all strata (< 65 bpm, p < 0.001; 65–75 bpm, p < 0.001; >75 bpm, p < 0.001) (**S1 Fig**). The association between the heart rate PRS and incident AF was not significant (<65 bpm, p = 0.027; 65–75 bpm, p = 0.017; >75 bpm, p = 0.293) (**S2 Fig**). Pleiotropy was assessed by performing regression analyses of resting heart rate PRS and incident AF, adjusted for resting heart rate (<65 bpm, p = 0.052; 65–75 bpm, p = 0.082; >75 bpm, p = 0.778) (**S3 Fig)**. Thus, the effect of the resting heart rate PRS on incident AF may be completely due to resting heart rate. Moreover, absence of pleiotropy was confirmed when the three p values of **S3 Fig** were combined using Fishers method (p = 0.076).

Causal effects, estimated by the ratio method of Mendelian randomization (**Fig 2**), showed an inverse relation between resting heart rate and incident AF (HR per 5 bpm, 0.82; 95% confidence interval (CI), 0.73–0.95; p = 0.010) for heart rates <65 bpm). The other strata of instrumental variable-free resting heart rates were not causally associated with incident AF (65–75 bpm, HR 0.82, 95% CI 0.59–1.05, p = 0.13; >75 bpm, HR 1.16, 95% CI 0.95–1.34, p = 0.15) (**Table 2**).

**Table 2:** Causal inference using instrumental variable analysis.

Stratum	Hazard ratio (per 5 bpm increase)	95% Confidence Interval		P-value
**Instrumental variable-free resting heart rate < 65 bpm**	0.82	0.73	0.95	0.010
**Instrumental variable-free resting heart rate ≥ 65 or < 75 bpm**	0.82	0.59	1.05	0.133
**Instrumental variable-free resting heart rate ≥ 75 bpm**	1.16	0.95	1.34	0.150

## Discussion

A causal association between resting heart rate and incident AF was investigated using Mendelian randomization analyses. Separate Mendelian randomization analyses were performed on three resting heart rate strata, because Mendelian randomization analyzes only linear associations. Results support an inverse causal association between genetically-determined resting heart rate and incident AF, for resting heart rates below 65 bpm. Despite suggestions of a U-shaped association between resting heart rate and incident AF, evidence for a causal U-shaped association could not be confirmed in our study.

Our study confirms that lower resting heart rates are associated with an increased risk of incident AF, as some studies demonstrated [9–11]. For example, in the Tromsᴓ Study, individuals with resting heart rates below 50 bpm had significantly increased risks of incident AF, compared to individuals with resting heart rates above 60 bpm [9]. Moreover, individuals exposed to years of endurance training are known to have a low resting heart rate and are reported to have increased risks of AF [20–23].

The mechanisms by which low resting heart rate may predispose to AF are poorly understood. On one hand, low resting heart rate is often associated with good exercise capacity and reduced morbidity and mortality [2]. However, low resting heart rates may indicate increased vagal tone. Enhanced vagal tone potentially creates conditions favouring excitability and atrial re-entry—via reduced effective refractory periods within the atria—leading to AF [20,24–26]. Our results suggest that low resting heart rate is causal to AF. Future studies may investigate whether low resting heart rates lead to changes in vagal tone, triggering AF.

An increased resting heart rate has also been proposed to be a marker for increased risks for AF [12,13]. Higher resting heart rates are associated with sympathetic overactivity and subclinical left ventricular dysfunction. Enhanced sympathetic tone may promote automaticity, reduce the atrial effective refractory period, and increase left atrial pressure via left ventricular dysfunction. These effects may induce AF [27–29]. However, our results provided causal evidence for only a reverse association between resting heart rate and AF, for resting heart rates below 65 bpm. In our data, prior evidence of an association between high resting heart rate and incident AF could not be translated to a causal interaction. Resting heart rates above 65 bpm may potentially be driven by other (sub)clinical comorbidities that increase sympathetic tone and are causal to incident AF. Although the results seem to show a U shaped trend, our data lack genetic support for a causal association between resting heart rates above 65 bpm and incident AF. Further research is needed to confirm.

Resting heart rate may be a useful component for understanding the complex mechanisms underlying the initiation of AF. Our Mendelian randomization study suggests a role for resting heart rate in the initiation of AF. Resting heart rate is easily measured in clinical practice and may be utilized for future AF risk assessments. Our results may support future studies that investigate low resting heart rate as a risk indicator or preventive measure of incident AF. The extent to which the epidemiological data of the U-shaped association reflect a causal association should be explored further.

### Strengths and limitations

Our study is one of the largest studies to specifically investigate causal evidence for the observed non-linear association between resting heart rate and incident AF. Moreover, the Mendelian randomization design is less susceptible to confounding, reverse causation, and selection bias, compared to observational analyses. However, several limitations should be discussed.

First, although the 10-second ECG is a standard method for measuring resting heart rate, unusual circumstances in combination with the short measurement time (10 seconds) can distort the resting heart rate.

Second, asymptomatic AF may have gone undetected in some individuals, leading to incorrect AF status (i.e., false negative classification). Considering the large number of participants, the effect of false-negative classification results may be limited.

Third, genetic variants associated with heart rate may have biased the heart rate PRS through (unknown) associations with AF or AF related risk factors. However, pleiotropic effects of heart rate were reduced to a minimum by adjusting the association between the heart rate PRS and incident AF for heart rate.

Fourth, information on negative dromotropic medication or pacemaker rhythm was not available for all participants. Individuals with higher heart rates may be more likely to be treated with heart rate lowering medications, which also reduces the risk of AF. Although participants with negative dromotropic medication or pacemaker rhythm were not excluded, the heart rate was still strongly associated with the constructed heart rate PRS (**S1 Fig**).

Fifth, the data of PROSPER showed a weaker association between heart rate and heart rate PRS, which may be due to the higher mean age of the population. Exclusion of individuals with myocardial infarction did not result in different data. However, the association between resting heart rate and heart rate PRS of all cohorts together was still significant.

Sixth, although our study is one of the largest studies to investigate causal evidence for the observed non-linear association between resting heart rate and incident AF, results could have been subjected to limited power. In the future more research is needed to confirm our conclusions.

Finally, the generalizability of our findings beyond European ancestry and the age range in the cohorts is uncertain.

## Conclusions

In seven cohorts of the AFGen consortium, genetically-determined resting heart rate was inversely associated with incident AF for resting heart rates below 65 bpm. Evidence for a causal U-shaped association could not be confirmed. Our results may suggest that low resting heart rate is not only a risk marker for AF, but may also cause incident AF. Further research is needed to elucidate the mechanisms underlying the association of low resting heart rate with AF.

## Supporting information

S1 Checklist (DOCX)

S2 Checklist (DOCX)

S1 File Genetic variants previously associated with resting heart rate used for Mendelian randomization. (DOCX)

S1 Fig Association of resting heart rate polygenic risk score and resting heart rate in the AFGen consortium. The results of a regression analyses of the Heart rate PRS and resting Heart rate is shown. Fig 1A shows the results of the regression performed in the strata with instrumental variable-free resting heart rate below 65 bpm (p<0.0001), Fig 1B shows the results of the regression performed in the strata with instrumental variable-free resting heart rate between 65 and 75 bpm (p<0.0001) and Fig 1C shows the results of the regression performed in the strata with instrumental variable-free resting heart rate of and above 75 bpm (p<0.0001). I^2^ reflects heterogeneity between studies, higher values reflect greater heterogeneity. Abbreviations: ARIC = Atherosclerosis Risk in Communities study, bpm = beats per minute, FHS = Framingham Heart Study, I^2^ = heterogeneity, MESA = Multi-Ethnic Study of Atherosclerosis, PREVEND = Prevention of Renal and Vascular End-stage Disease study, PROSPER = PROspective Study of Pravastatin in the Elderly at Risk study, PRS = polygenic risk score, RS = Rotterdam Study, se = standard error of the effect size, SHIP = Study of Health in Pomerania, t^2^ = between study variance.
(PNG)

S2 Fig Association of resting heart rate polygenic risk score and incident AF in the AFGen consortium. The results of a regression analyses of the Heart rate PRS and incident AF is shown. Fig 2A shows the results of the regression performed in the strata with instrumental variable-free resting heart rate below 65 bpm of the strata (p = 0.027), Fig 2B shows the results of the regression performed in the strata with instrumental variable-free resting heart rate between 65 and 75 bpm (p = 0.017) and Fig 2C shows the results of the regression performed in the strata with instrumental variable-free resting heart rate of and above 75 bpm (p = 0.29). I^2^ reflects heterogeneity between studies, higher values reflect greater heterogeneity. Abbreviations: ARIC = Atherosclerosis Risk in Communities study, bpm = beats per minute, FHS = Framingham Heart Study, I^2^ = heterogeneity, MESA = Multi-Ethnic Study of Atherosclerosis, PREVEND = Prevention of Renal and Vascular End-stage Disease study, PROSPER = PROspective Study of Pravastatin in the Elderly at Risk study, PRS = polygenic risk score, RS = Rotterdam Study, se = standard error of the effect size, SHIP = Study of Health in Pomerania, t^2^ = between study variance.
(PNG)

S3 Fig Association of resting heart rate polygenic risk score and incident AF adjusted for resting heart rate in the AFGen consortium. The results of a regression analyses of the Heart rate PRS and incident AF adjusted for heart rate is shown. The Heart rate PRS should not be associated with incident AF if adjusted for heart rate; this would be evidence for pleiotropic effects of the Heart rate PRS. Fig 3A shows the results of the regression performed in the strata with instrumental variable-free resting heart rate below 65 bpm of the strata (p = 0.052), Fig 3B shows the results of the regression performed in the strata with instrumental variable-free resting heart rate between 65 and 75 bpm (p = 0.111), and Fig 3C shows the results of the regression performed in the strata with instrumental variable-free resting heart rate of and above 75 bpm (p = 0.778). I^2^ reflects heterogeneity between studies, higher values reflect greater heterogeneity. Abbreviations: ARIC = Atherosclerosis Risk in Communities study, bpm = beats per minute, FHS = Framingham Heart Study, I^2^ = heterogeneity, MESA = Multi-Ethnic Study of Atherosclerosis, PREVEND = Prevention of Renal and Vascular End-stage Disease study, PROSPER = PROspective Study of Pravastatin in the Elderly at Risk study, PRS = polygenic risk score, RS = Rotterdam Study, se = standard error of the effect size, SHIP = Study of Health in Pomerania, t^2^ = between study variance.
(PNG)
