# Supplementary material for: Resting heart rate and incident atrial fibrillation: A stratified Mendelian randomization in the AFGen consortium
Source: PLoS One. 2022 May 20;17(5):e0268768. doi: 10.1371/journal.pone.0268768 (PMC9122202; doi:10.1371/journal.pone.0268768)
Supplement: S1 File — (DOCX) [file pone.0268768.s003.docx]

# Supplementary information

## Table of contents

**S1 Table.** Genetic variants previously associated with resting heart rate used for Mendelian randomization.

**Figure Legends**

**S1 Fig.** Association of resting heart rate polygenic risk score and resting heart rate in the AFGen consortium.

**S2 Fig.** Association of resting heart rate polygenic risk score and incident AF in the AFGen consortium.

**S3 Fig.** Association of resting heart rate polygenic risk score and incident AF adjusted for resting heart rate in the AFGen consortium.

**Supplementary notes** Description of included cohorts
**Sources of Funding** Description of funding

**References**

**S1 Table. Genetic variants used for Mendelian randomization.**

| **Variant** | **Non-coded Allele** | **Coded Allele** | **Beta-coefficient** |
| --- | --- | --- | --- |
| rs151041685 | G | T | 1.061 |
| rs182770070 | A | T | 0,94 |
| rs1320761 | C | T | 0,902 |
| rs422068 | T | C | 0,731 |
| rs11183443 | T | C | 0,676 |
| rs41317993 | G | A | 0,63 |
| rs17881696 | G | A | 0,578 |
| rs3951016 | T | A | 0,52 |
| rs11320420 | GA | G | 0,427 |
| rs12889267 | A | G | 0,416 |
| rs117159291 | A | C | 0,404 |
| rs1994135 | T | C | 0,4 |
| rs174536; (proxy of rs11320420 - R2=0,96) | A | C | 0,399 |
| rs73158705 | A | G | 0,393 |
| rs272564 | A | C | 0,351 |
| rs62172372 | A | G | 0,337 |
| rs3915499 | G | A | 0,303 |
| rs867400 | T | C | 0,298 |
| rs12501032 | C | G | 0,288 |
| rs1483890 | A | G | 0,284 |
| rs236349 | A | G | 0,281 |
| rs11920570 | G | A | 0,268 |
| rs3749237 | G | A | 0,258 |
| rs11454451 | C | CT | 0,256 |
| rs12576326 | A | G | 0,253 |
| rs4608502 | T | C | 0,249 |
| rs16974196 | G | A | 0,244 |
| rs2744375 | A | T | 0,24 |
| rs17265513 | T | C | 0,24 |
| rs62144050 | T | C | 0,225 |
| rs10880689; (proxy of rs11183443 - R2=0,92) | A | G | 0,208 |
| rs1549118 | C | T | 0,2 |
| rs4900069 | A | C | 0,2 |
| rs2283847 | C | T | -0,174 |
| rs12941356 | A | G | -0,181 |
| rs1425518 | C | T | -0,182 |
| rs1592560 | A | C | -0,183 |
| rs17494056 | A | C | -0,184 |
| rs10820614 | G | C | -0,185 |
| rs41748 | T | G | -0,193 |
| rs12713404 | G | T | -0,199 |
| rs748802 | G | A | -0,202 |
| rs2358740 | G | T | -0,208 |
| rs1050288 | C | T | -0,213 |
| rs13165531 | A | T | -0,221 |
| rs11563648 | G | C | -0,231 |
| rs10841486 | T | C | -0,238 |
| rs58437978 | T | C | -0,24 |
| rs12579753 | C | T | -0,246 |
| rs1468333; (proxy of rs35284930 - R2=0,85) | T | C | -0,255 |
| rs145358377 | G | GA | -0,259 |
| rs10739663 | A | G | -0,266 |
| rs11081761 | G | A | -0,267 |
| rs1260326 | T | C | -0,275 |
| rs11083258 | A | C | -0,276 |
| rs12721051 | C | G | -0,287 |
| rs7194801 | T | C | -0,291 |
| rs35284930 | GA | G | -0,293 |
| rs2076028 | G | A | -0,295 |
| rs2152735 | G | A | -0,306 |
| rs41312411 | C | G | -0,32 |
| rs180239 | G | C | -0,326 |
| rs13002735 | A | C | -0,331 |
| rs138186803 | AT | A | -0,333 |
| rs907683 | G | T | -0,334 |
| rs6845865 | T | C | -0,342 |
| rs564190295 | G | GCCGCCGCCCCC | -0,355 |
| rs4868243 | G | A | -0,361 |
| rs2283274 | G | C | -0,405 |
| rs17201923 | A | G | -0,41 |
| rs7612445 | G | T | -0,428 |
| rs79121763 | C | T | -0,471 |
| rs17180489 | G | C | -0,49 |
| rs75190942 | C | A | -0,496 |
| rs7173389 | A | T | -0,539 |
| rs6123471 | T | C | -0,595 |
| rs56233017 | G | A | -0,666 |
| rs4963772 | G | A | -0,714 |
| rs61735998 | G | T | -0,834 |

# Figure legends

**S1 Fig. Association of resting heart rate polygenic risk score and resting heart rate in the AFGen consortium.** The results of a regression analyses of the Heart rate PRS and resting Heart rate is shown. Figure 1A shows the results of the regression performed in the strata with instrumental variable-free resting heart rate below 65 bpm (p<0.0001), figure 1B shows the results of the regression performed in the strata with instrumental variable-free resting heart rate between 65 and 75 bpm (p<0.0001) and figure 1C shows the results of the regression performed in the strata with instrumental variable-free resting heart rate of and above 75 bpm (p<0.0001). I^2^ reflects heterogeneity between studies, higher values reflect greater heterogeneity. Abbreviations: ARIC = Atherosclerosis Risk in Communities study, bpm = beats per minute, FHS = Framingham Heart Study, I^2^ = heterogeneity, MESA= Multi-Ethnic Study of Atherosclerosis, PREVEND = Prevention of Renal and Vascular End-stage Disease study, PROSPER = PROspective Study of Pravastatin in the Elderly at Risk study, PRS = polygenic risk score, RS = Rotterdam Study, se = standard error of the effect size, SHIP = Study of Health in Pomerania, t^2^ = between study variance.

**S2 Fig. Association of resting heart rate polygenic risk score and incident AF in the AFGen consortium.** The results of a regression analyses of the Heart rate PRS and incident AF is shown. Figure 2A shows the results of the regression performed in the strata with instrumental variable-free resting heart rate below 65 bpm of the strata (p=0.027), figure 2B shows the results of the regression performed in the strata with instrumental variable-free resting heart rate between 65 and 75 bpm (p=0.017) and figure 2C shows the results of the regression performed in the strata with instrumental variable-free resting heart rate of and above 75 bpm (p=0.29). I^2^ reflects heterogeneity between studies, higher values reflect greater heterogeneity. Abbreviations: ARIC = Atherosclerosis Risk in Communities study, bpm = beats per minute, FHS = Framingham Heart Study, I^2^ = heterogeneity, MESA = Multi-Ethnic Study of Atherosclerosis, PREVEND = Prevention of Renal and Vascular End-stage Disease study, PROSPER = PROspective Study of Pravastatin in the Elderly at Risk study, PRS = polygenic risk score, RS = Rotterdam Study, se = standard error of the effect size, SHIP = Study of Health in Pomerania, t^2^ = between study variance.

**S3 Fig. Association of resting heart rate polygenic risk score and incident AF adjusted for resting heart rate in the AFGen consortium.** The results of a regression analyses of the Heart rate PRS and incident AF adjusted for heart rate is shown. The Heart rate PRS should not be associated with incident AF if adjusted for heart rate; this would be evidence for pleiotropic effects of the Heart rate PRS. Figure 3A shows the results of the regression performed in the strata with instrumental variable-free resting heart rate below 65 bpm of the strata (p=0.052), figure 3B shows the results of the regression performed in the strata with instrumental variable-free resting heart rate between 65 and 75 bpm (p=0.111), and figure 3C shows the results of the regression performed in the strata with instrumental variable-free resting heart rate of and above 75 bpm (p=0.778). I^2^ reflects heterogeneity between studies, higher values reflect greater heterogeneity. Abbreviations: ARIC = Atherosclerosis Risk in Communities study, bpm = beats per minute, FHS = Framingham Heart Study, I^2^ = heterogeneity, MESA = Multi-Ethnic Study of Atherosclerosis, PREVEND = Prevention of Renal and Vascular End-stage Disease study, PROSPER = PROspective Study of Pravastatin in the Elderly at Risk study, PRS = polygenic risk score, RS = Rotterdam Study, se = standard error of the effect size, SHIP= Study of Health in Pomerania, t^2^ = between study variance.

**S1 Fig. Association of resting heart rate polygenic risk score and resting heart rate in the AFGen consortium.**


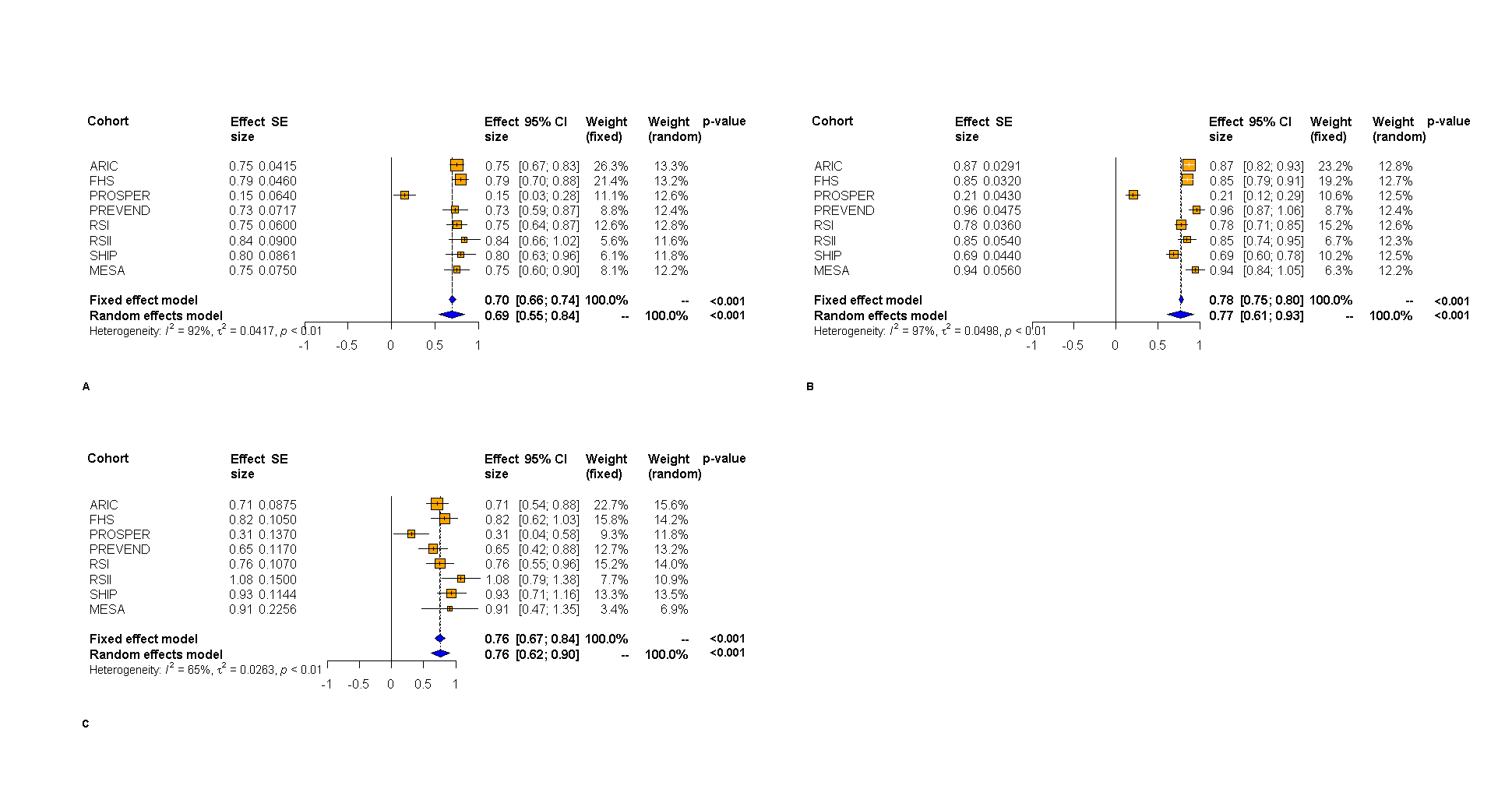


**S2 Fig. Association of resting heart rate polygenic risk score and incident AF in the AFGen consortium.**

**
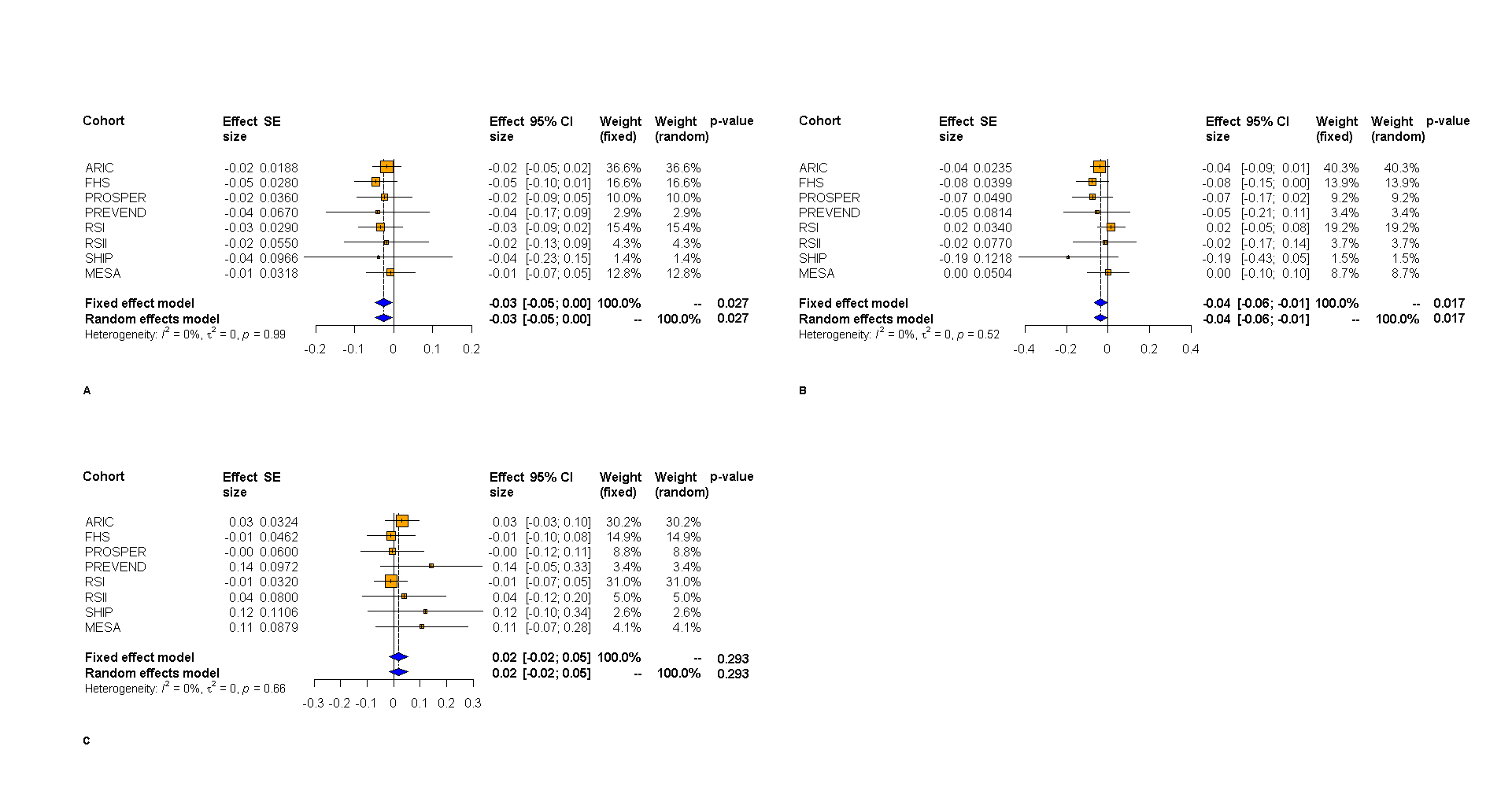
**

**S3 Fig. Association of resting heart rate polygenic risk score and incident AF adjusted for resting heart rate in the AFGen consortium.
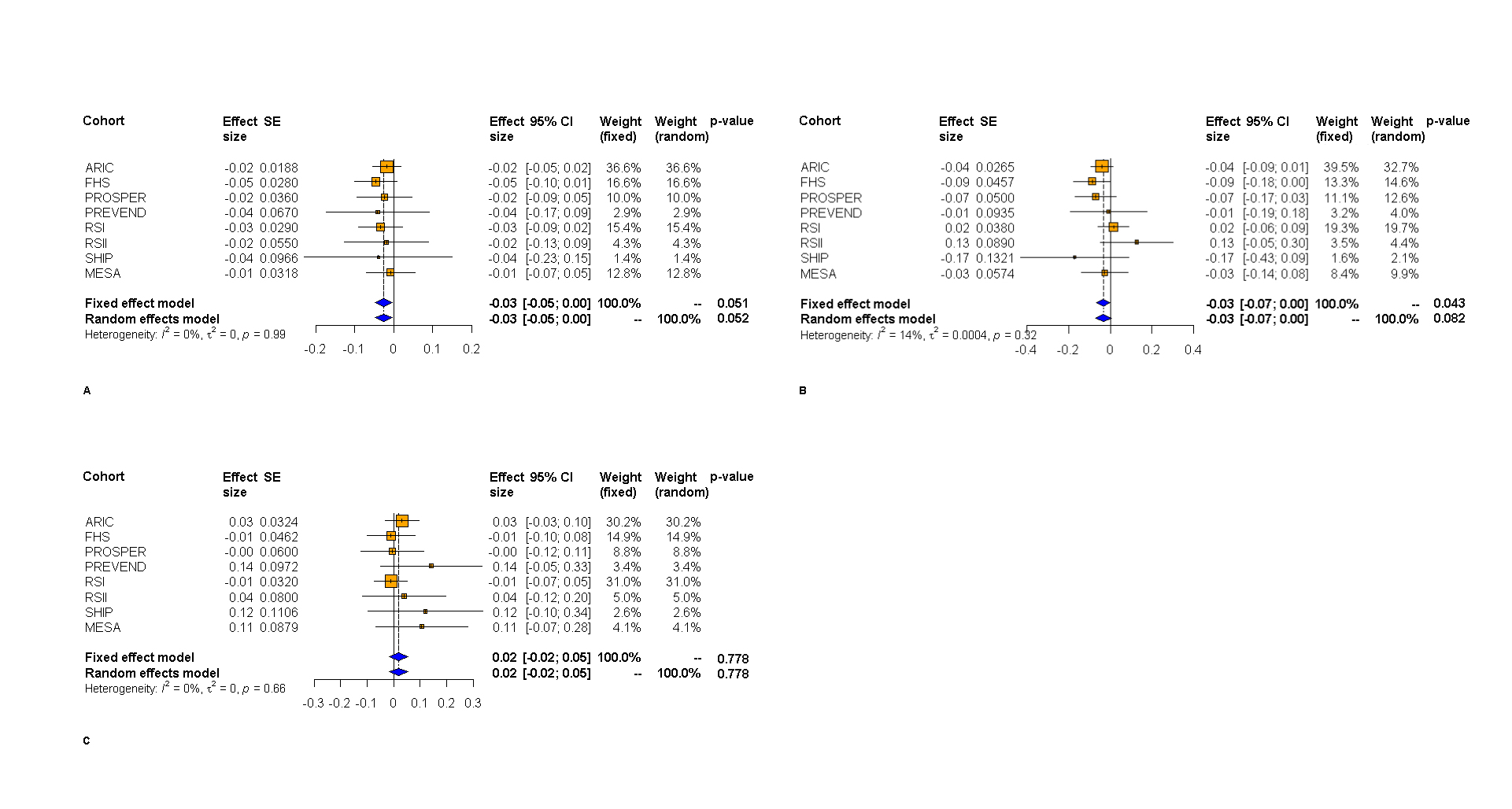
**

# Supplementary notes

**Description of included cohorts**

**The following cohorts were described previously:** The Framingham Heart Study (FHS)^1^ and the Prevention of Renal and Vascular End-stage Disease (PREVEND) study.^2^

**The Atherosclerosis Risk in Communities study.** Atherosclerosis Risk in Communities (ARIC) is a prospective population-based study of subjects in the United States (73% of European descent) aged 45 to 64 years at enrollment, recruited from four US communities (suburbs of Minneapolis, Minnesota; Washington County, Maryland; Jackson, Mississippi; and Forsyth County, North Carolina) between 1987-1989 to investigate the epidemiology of cardiovascular disease. Participants underwent electrocardiograms at baseline and at each follow-up exam (3 exams; 1 exam every 3 years). Incident atrial fibrillation was classified as the first occurrence of atrial fibrillation through 2005 as identified from electrocardiograms at study visits, hospital discharge codes or death certificates (ICD-9 code 427.31 or 427.32, or ICD-10 code I48). The sensitivity and positive predictive value of hospital discharge codes for the diagnosis of incident atrial fibrillation, as determined after review of hospital discharge summaries in a sample of ARIC participants, was close to 90%. Only subjects of self-reported European ancestry were included in this analysis; thus, subjects recruited from Jackson, MS, and a small group of subjects from Forsyth County, NC, were not included.

**The Multi-Ethnic Study of Atherosclerosis.** The Multi-Ethnic Study of Atherosclerosis (MESA) is a study of the characteristics of subclinical cardiovascular disease (disease detected non-invasively before it has produced clinical signs and symptoms) and the risk factors that predict progression to clinically overt cardiovascular disease or progression of the subclinical disease. The cohort is a diverse, population-based sample of 6,814 asymptomatic men and women aged 45-84. Approximately 38 percent of the recruited participants are white, 28 percent African-American, 22 percent Hispanic, and 12 percent Asian (predominantly of Chinese descent). Participants were recruited during 2000-2002 from 6 field centers across the U.S. (at Wake Forest University; Columbia University; Johns Hopkins University; the University of Minnesota; Northwestern University; and the University of California – Los Angeles). All underwent anthropomorphic measurement and extensive evaluation by questionnaires at baseline. Six exams have been completed since 2000. Participants are contacted every 9 to 12 months throughout the study to assess clinical morbidity and mortality. Further information can be found at: <http://www.ncbi.nlm.nih.gov/projects/gap/cgi-bin/study.cgi?study_id=phs000209.v13.p3> and [http://www.mesa-nhlbi.org](https://eur03.safelinks.protection.outlook.com/?url=http%3A%2F%2Fwww.mesa-nhlbi.org%2F&data=02%7C01%7Cj.e.siland%40umcg.nl%7C36fc03edf58e4e3f72bf08d80824a5a1%7C335122f9d4f44d67a2fccd6dc20dde70%7C0%7C0%7C637268301688517367&sdata=hOcpMwSmNPcGJRum0iUzRJOSTpNAQyOJoA2Qba98Q%2Fs%3D&reserved=0).

**The PROspective Study of Pravastatin in the Elderly at Risk**. All data come from the PROspective Study of Pravastatin in the Elderly at Risk (PROSPER). A detailed description of the study has been published elsewhere. PROSPER was a prospective multicenter randomized placebo-controlled trial to assess whether treatment with pravastatin diminishes the risk of major vascular events in elderly. Between December 1997 and May 1999, we screened and enrolled subjects in Scotland (Glasgow), Ireland (Cork), and the Netherlands (Leiden). Men and women aged 70-82 years were recruited if they had pre-existing vascular disease or increased risk of such disease because of smoking, hypertension, or diabetes. A total number of 5,804 subjects were randomly assigned to pravastatin or placebo. A large number of prospective tests were performed including Biobank tests and cognitive function measurements. A whole genome wide screening has been performed in the sequential PHASE project. Of 5,763 subjects DNA was available for genotyping. Genotyping was performed with the Illumina 660K beadchip, after QC (call rate <95%) 5,244 subjects and 557,192 SNPs were left for analysis. These SNPs were imputed to 2.5 million SNPs based on the HAPMAP built 36 with MACH imputation software. The study was approved by the institutional ethics review boards of centers of Cork University (Ireland), Glasgow University (Scotland) and Leiden University Medical Center (the Netherlands) and all participants gave written informed consent.

**Rotterdam Study.** The Rotterdam Study was designed as a prospective cohort study (RS I), initially comprising 7983 persons of 55 years or older, living in the well-defined Ommoord district in the city of Rotterdam in The Netherlands (78% of 10,215 invitees). From January 1990 onwards participants were recruited for the Rotterdam Study. In 2000, 3011 participants (out of 4472 invitees) who had become 55 years of age or moved into the study district since the start of the study were added to the cohort (RS II). In 2006, a further extension of the cohort was initiated in which 3932 subjects were included, aged 45–54 years, out of 6057 invited, living in the Ommoord district (RS III). By the end of 2008, the Rotterdam Study therefore comprised 14,926 subjects aged 45 years or over. The overall response figure for all three cycles at baseline was 72.0% (14, 926 out of 20, 744).
The participants were all extensively examined at study entry (i.e. baseline) and subsequent follow-up visits that take place every 3 to 6 years. They were interviewed at home (2 h) and then underwent an extensive set of examinations (a total of 5 h) in a specially built research facility in the centre of the district. These examinations focused on possible causes of invalidating diseases in the elderly in a clinically state-of-the-art manner, as far as the circumstances allowed. The emphasis was put on imaging (of heart, blood vessels, eyes, skeleton and later brain) and on collecting biospecimens that enabled further in-depth molecular and genetic analyses.
The participants in the Rotterdam Study are followed for a variety of diseases that are frequent in the elderly, which include but are not exclusive to coronary heart disease, heart failure and stroke, Parkinson disease, Alzheimer disease and other dementias, depression and anxiety disorders, macular degeneration and glaucoma, COPD, emphysema, liver diseases, diabetes mellitus, osteoporosis, dermatological diseases and cancer. There is a complete coverage of filled prescriptions and there are repeated interviews of medicines use.
The Rotterdam Study has been approved by the Medical Ethics Committee of the Erasmus MC (registration number MEC 02.1015) and by the Dutch Ministry of Health, Welfare and Sport (Population Screening Act WBO, license number 1071272-159521-PG).

**The Study of Health in Pomerania.** The Study of Health in Pomerania (SHIP) is a prospective longitudinal population-based cohort study in Western Pomerania assessing the prevalence and incidence of common diseases and their risk factors. Participants aged 20 to 79 with German citizenship and principal residency in the study area were recruited from a random sample of residents living in the three local cities, 12 towns as well as 17 randomly selected smaller towns. Individuals were randomly selected stratified by age and sex in proportion to population size of the city, town or small towns, respectively. A total of 4,308 participants were recruited between 1997 and 2001 in the SHIP cohort. Individuals were invited to the SHIP study centre for a computer-assisted personal interviews and extensive physical examinations.

Description of funding
The Atherosclerosis Risk in Communities study has been funded in whole or in part with funds from the National Heart, Lung, and Blood Institute, National Institutes of Health, Department of Health and Human Service (contract numbers HHSN268201700001I, HHSN268201700002I, HHSN268201700003I, HHSN268201700005I, and HHSN268201700004I, R01HL087641, R01HL059367, and R01HL086694; National Human Genome Research Institute contract U01HG004402; and National Institutes of Health contract HHSN268200625226C). Additional support for this analysis was provided by NHLBI grant K24HL148521 and American Heart Association grant 16EIA26410001. The authors thank the staff and participants of the ARIC study for their important contributions.

The Framingham Heart Study is conducted and supported by the National Heart, Lung, and Blood Institute (NHLBI) in collaboration with Boston University (contract nos. N01-HC-25195, HHSN268201500001I, and 75N92019D00031). Dr. Kornej was supported by Marie Sklodowska-Curie Actions under the European Union’s Horizon 2020 research and innovation programme (grant agreement no. 838259). Dr. Benjamin is supported by NIH NHLBI grants R01HL092577 and 1R01HL128914 and American Heart Association 18SFRN34110082. Dr. Lubitz is supported by NIH grant 1R01HL139731 and American Heart Association 18SFRN34250007. Dr. Lubitz receives sponsored research support from Bristol Myers Squibb / Pfizer, Bayer AG, Boehringer Ingelheim, Fitbit, and IBM, and has consulted for Bristol Myers Squibb / Pfizer, Bayer AG, and Blackstone Life Sciences.

MESA and the MESA SHARe project are conducted and supported by the National Heart, Lung, and Blood Institute (NHLBI) in collaboration with MESA investigators. Support for MESA is provided by contracts 75N92020D00001, HHSN268201500003I, N01-HC-95159, 75N92020D00005, N01-HC-95160, 75N92020D00002, N01-HC-95161, 75N92020D00003, N01-HC-95162, 75N92020D00006, N01-HC-95163, 75N92020D00004, N01-HC-95164, 75N92020D00007, N01-HC-95165, N01-HC-95166, N01-HC-95167, N01-HC-95168, N01-HC-95169, UL1-TR-000040, UL1-TR-001079, UL1-TR-001420, UL1-TR-001881, and DK063491. Funding for SHARe genotyping was provided by NHLBI contract N02-HL-64278. Genotyping was performed at Affymetrix (Santa Clara, California, USA) and the Broad Institute of Harvard and MIT (Boston, Massachusetts, USA) using the Affymetrix Genome-Wide Human SNP Array 6.0.

PREVEND is supported by the Dutch Kidney Foundation (grant E0.13) and the Netherlands Heart Foundation (grant NHS2010B280).

The PROSPER study was supported by an investigator-initiated grant obtained from Bristol-Myers Squibb. Prof. Dr. J. W. Jukema is an Established Clinical Investigator of the Netherlands Heart Foundation (grant 2001 D 032). Support for genotyping was provided by the seventh framework program of the European commission (grant 223004) and by the Netherlands Genomics Initiative (Netherlands Consortium for Healthy Aging grant 050-060-810).

The Rotterdam Study is supported by the Erasmus University Medical Center and Erasmus University, Rotterdam; The Netherlands Organisation for Scientific Research (NWO); The Netherlands Organisation for Health Research and Development (ZonMw); the Research Institute for Diseases in the Elderly (RIDE); The Netherlands Genomics Initiative (NGI); the Ministry of Education, Culture and Science; the Ministry of Health, Welfare and Sports; the European Commission (DG XII); and the Municipality of Rotterdam. The contribution of inhabitants, general practitioners and pharmacists of the Ommoord district to the Rotterdam Study is gratefully acknowledged.

The Study of Health in Pomerania (SHIP) is supported by the German Federal Ministry of Education and Research (Bundesministerium für Bildung und Forschung (BMBF); grants 01ZZ9603, 01ZZ0103, and 01ZZ0403) and the German Research Foundation (Deutsche Forschungsgemeinschaft (DFG); grant GR 1912/5-1). SHIP is part of the Community Medicine Research net (CMR) of the University of Greifswald which is funded by the BMBF as well as the Ministry for Education, Science and Culture and the Ministry of Labor, Equal Opportunities, and Social Affairs of the Federal State of Mecklenburg-West Pomerania. The CMR encompasses several research projects that share data from SHIP. Genome-wide data have been supported by a joint grant from Siemens Healthcare, Erlangen, Germany and the Federal State of Mecklenburg-West Pomerania.

The AFGen consortium is supported by R01HL128914 and 2R01 HL092577 and American Heart Association grant 18SFRN34110082.

# References

1. Ellinor PT, Lunetta KL, Albert CM, et al. Meta-analysis identifies six new susceptibility loci for atrial fibrillation. *Nat Genet*. 2012;44(6):670-675.

2. Christophersen IE, Rienstra M, Roselli C, et al. Large-scale analyses of common and rare variants identify 12 new loci associated with atrial fibrillation. *Nat Genet*. 2017;49(6):946-952.
