# Supplementary figures and images for: Resting heart rate and incident atrial fibrillation: A stratified Mendelian randomization in the AFGen consortium
Source: PLoS One. 2022 May 20;17(5):e0268768. doi: 10.1371/journal.pone.0268768 (PMC9122202; doi:10.1371/journal.pone.0268768)

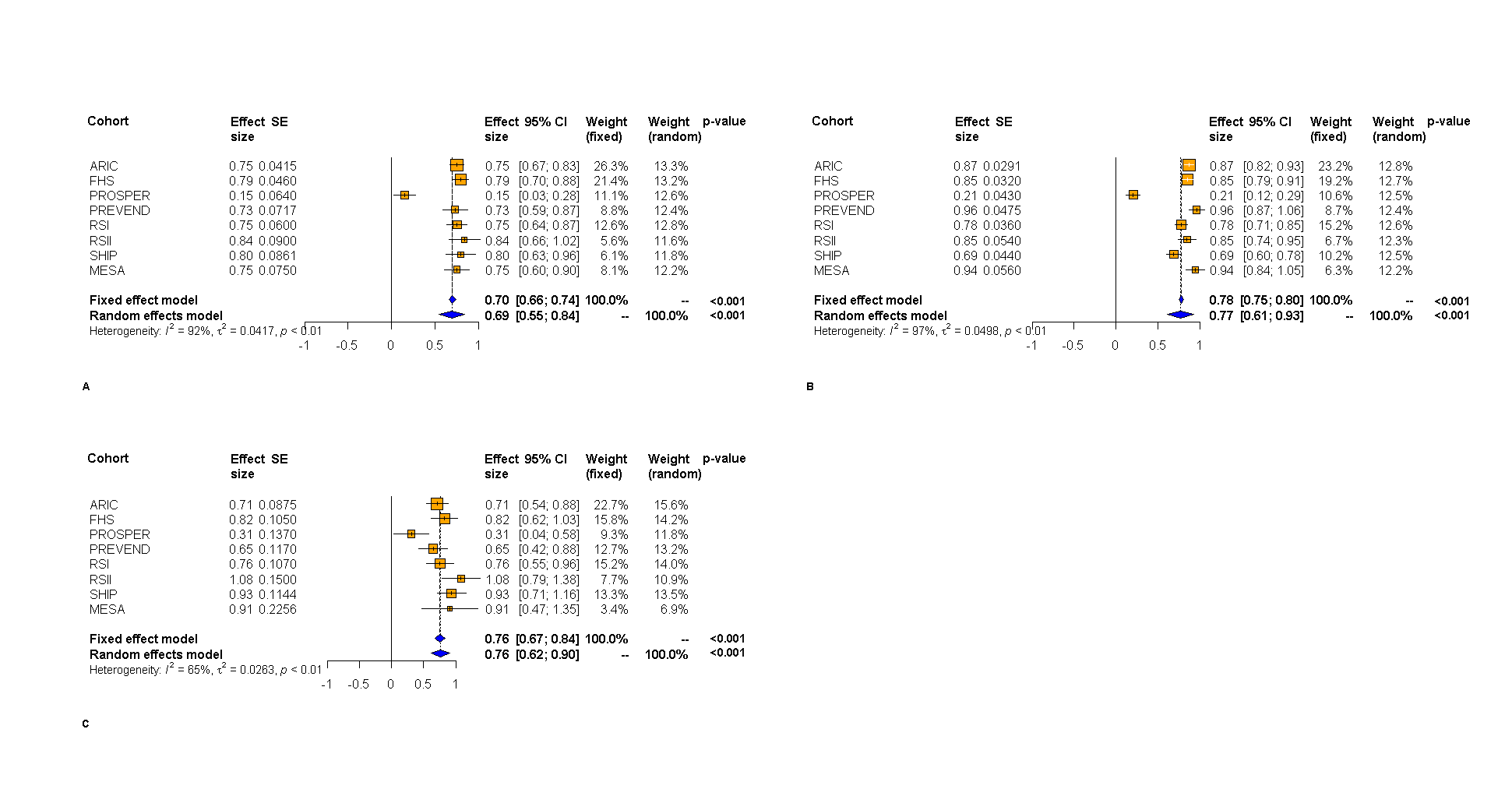

Supplement: S1 Fig — The results of a regression analyses of the Heart rate PRS and resting Heart rate is shown. Fig 1A shows the results of the regression performed in the strata with instrumental variable-free resting heart rate below 65 bpm (p<0.0001), Fig 1B shows the results of the regression performed in the strata with instrumental variable-free resting heart rate between 65 and 75 bpm (p<0.0001) and Fig 1C shows the results of the regression performed in the strata with instrumental variable-free resting heart rate of and above 75 bpm (p<0.0001). I2 reflects heterogeneity between studies, higher values reflect greater heterogeneity. Abbreviations: ARIC = Atherosclerosis Risk in Communities study, bpm = beats per minute, FHS = Framingham Heart Study, I2 = heterogeneity, MESA = Multi-Ethnic Study of Atherosclerosis, PREVEND = Prevention of Renal and Vascular End-stage Disease study, PROSPER = PROspective Study of Pravastatin in the Elderly at Risk study, PRS = polygenic risk score, RS = Rotterdam Study, se = standard error of the effect size, SHIP = Study of Health in Pomerania, t2 = between study variance. (PNG) [file pone.0268768.s004.png]

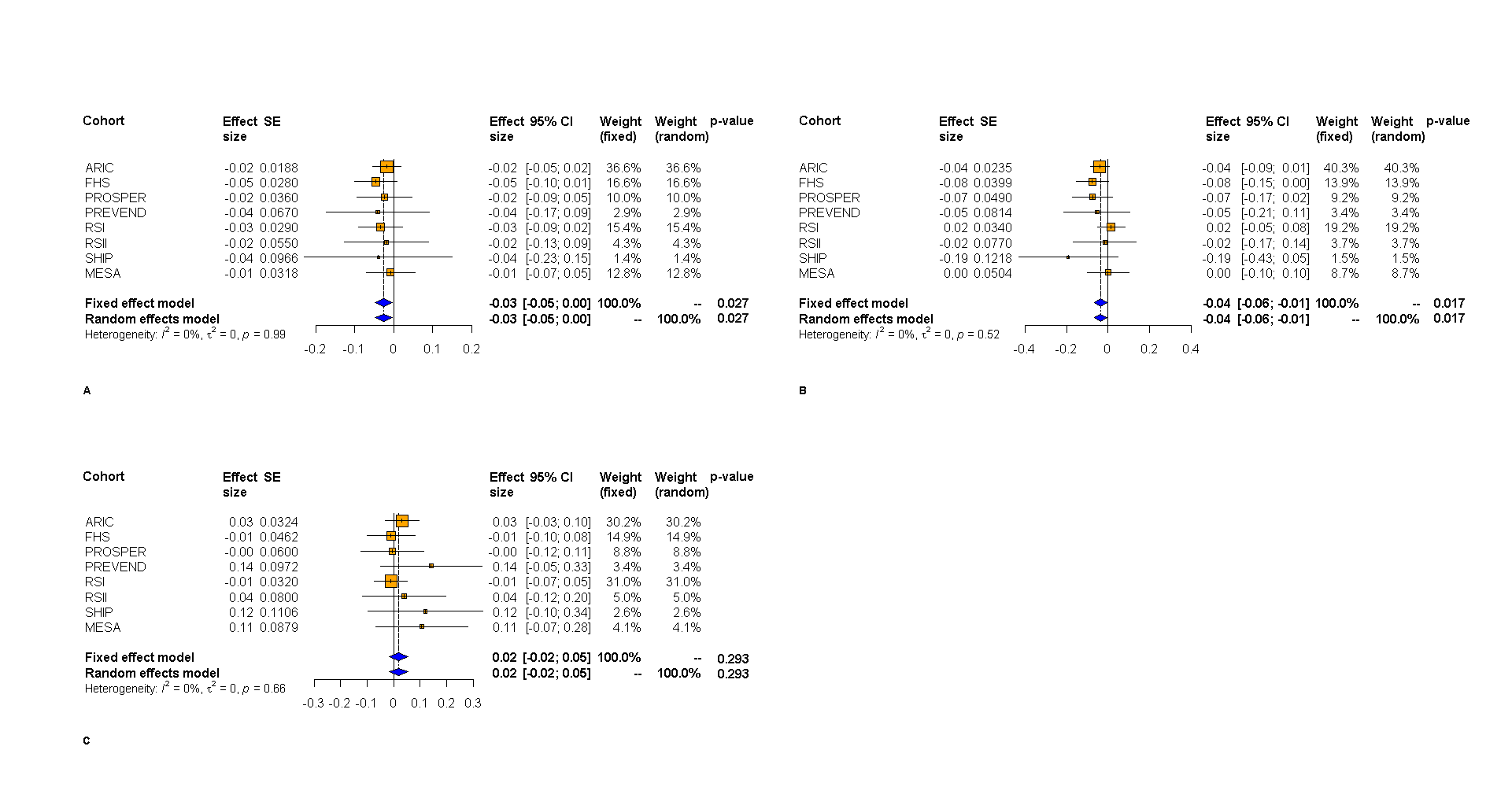

Supplement: S2 Fig — The results of a regression analyses of the Heart rate PRS and incident AF is shown. Fig 2A shows the results of the regression performed in the strata with instrumental variable-free resting heart rate below 65 bpm of the strata (p = 0.027), Fig 2B shows the results of the regression performed in the strata with instrumental variable-free resting heart rate between 65 and 75 bpm (p = 0.017) and Fig 2C shows the results of the regression performed in the strata with instrumental variable-free resting heart rate of and above 75 bpm (p = 0.29). I2 reflects heterogeneity between studies, higher values reflect greater heterogeneity. Abbreviations: ARIC = Atherosclerosis Risk in Communities study, bpm = beats per minute, FHS = Framingham Heart Study, I2 = heterogeneity, MESA = Multi-Ethnic Study of Atherosclerosis, PREVEND = Prevention of Renal and Vascular End-stage Disease study, PROSPER = PROspective Study of Pravastatin in the Elderly at Risk study, PRS = polygenic risk score, RS = Rotterdam Study, se = standard error of the effect size, SHIP = Study of Health in Pomerania, t2 = between study variance. (PNG) [file pone.0268768.s005.png]

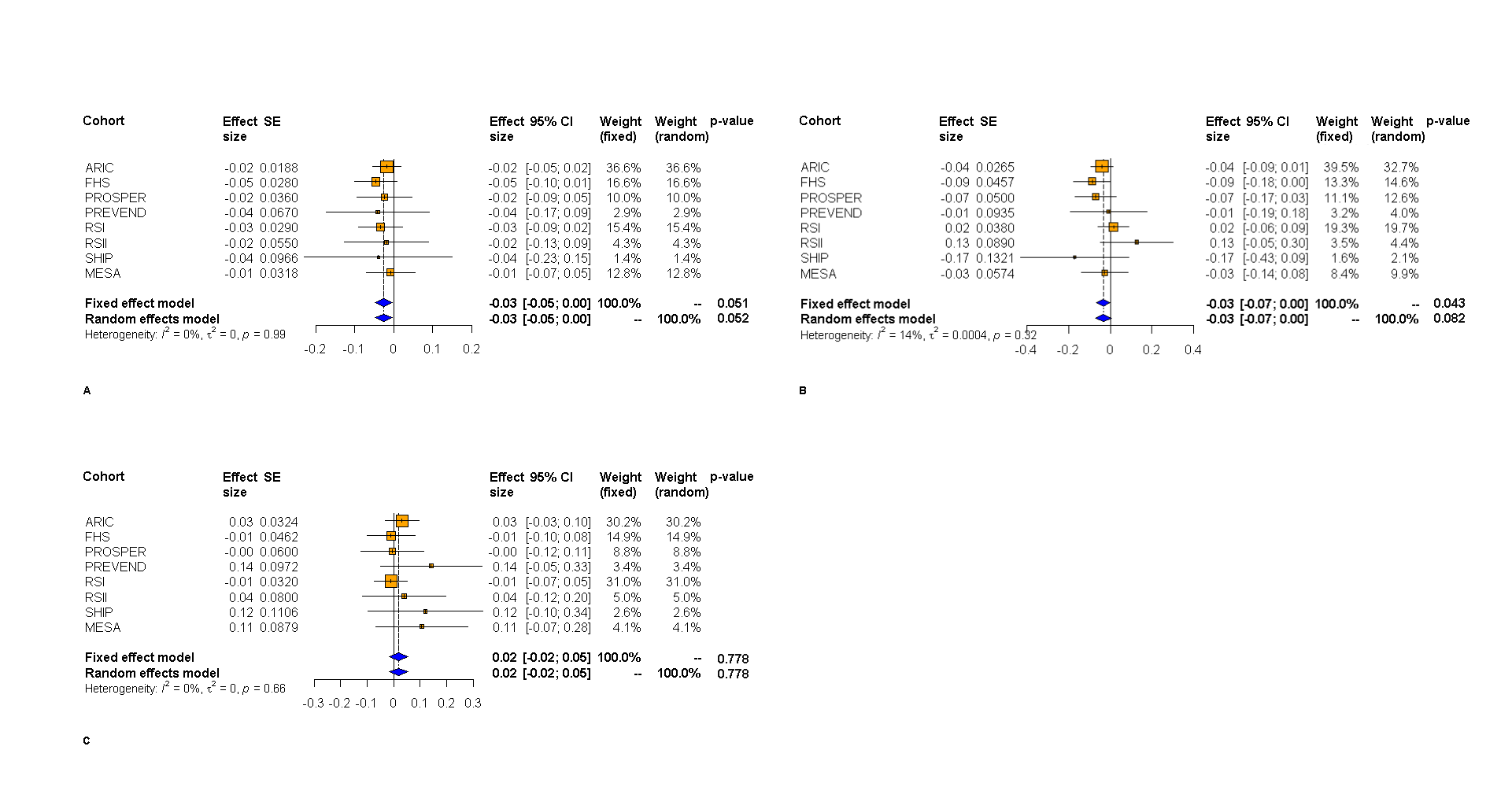

Supplement: S3 Fig — The results of a regression analyses of the Heart rate PRS and incident AF adjusted for heart rate is shown. The Heart rate PRS should not be associated with incident AF if adjusted for heart rate; this would be evidence for pleiotropic effects of the Heart rate PRS. Fig 3A shows the results of the regression performed in the strata with instrumental variable-free resting heart rate below 65 bpm of the strata (p = 0.052), Fig 3B shows the results of the regression performed in the strata with instrumental variable-free resting heart rate between 65 and 75 bpm (p = 0.111), and Fig 3C shows the results of the regression performed in the strata with instrumental variable-free resting heart rate of and above 75 bpm (p = 0.778). I2 reflects heterogeneity between studies, higher values reflect greater heterogeneity. Abbreviations: ARIC = Atherosclerosis Risk in Communities study, bpm = beats per minute, FHS = Framingham Heart Study, I2 = heterogeneity, MESA = Multi-Ethnic Study of Atherosclerosis, PREVEND = Prevention of Renal and Vascular End-stage Disease study, PROSPER = PROspective Study of Pravastatin in the Elderly at Risk study, PRS = polygenic risk score, RS = Rotterdam Study, se = standard error of the effect size, SHIP = Study of Health in Pomerania, t2 = between study variance. (PNG) [file pone.0268768.s006.png]
